# Impulse of the Healthy Heart

**Published:** 1850-07

**Authors:** O’Bryen Bellingham


### RECORD OF MEDICAL SCIENCE.

### ANATOMY AND PHYSIOLOGY.

*Impulse of the healthy heart.* By O'Bryen Bellingham, M. D.—The impulse of the heart accompanies the sys ole of the ventricles and the first sound of the organ, and has its cause in the apex of the heart coming in contact with the parietes of the thorax between the cartilages of the fifth and sixth left ribs. The mechanism by which the impulse is produced, was long a disputed point, and various have been the theories advanced by physiologists to explain it: even the period of the heart's action at which it occurs has been the subject of difference of opinion.

Thus it was at one time maintained that the impulse occurred during the diastole of the ventricles; and this opinion appeared to derive confirmation from the fact that when the heart of the frog is exposed (which will continue to pulsate for a considerable time after being laid bare,) the ventricle, during its diastole, is seen to expand, and to approach the parietes; while during the systole the apex is simply approximated to the base. In this animal, therefore, the heart approaches the parietes during the diastole, not during the systole of the ventricle, and any impulse which is given, must be at the period of the ventricular systole. An experiment was performed by Oesterreicher, which consisted in removing the heart of the frog from the body, and laying upon it a substance sufficiently heavy to press it flat, and yet so small as not to conceal the heart from view. He states that during the systole of the ventricle the weight was raised, but that during its diastole the heart remained flat. This experiment has been quoted by Muller and others, to prove that the diastole of the ventricles is not a muscular act, in ignorance, apparently, of the foregoing peculiarity in the action of the heart in this animal. In warm blooded animals, however, experiments and observations repeated over and over again have proved that the impulse occurs at the period of the ventricular systole, and that it is due to the apex of the heart coming in contact with the parietes of the chest.

*Mechanism by which the impulse of the heart is produced.*—It will not be necessary to delay to notice the various theories which have been advanced in order to explain the mechanism by which the impulse of the heart is produced: the majority of these are founded on erroneous views. It will be sufficient to observe, that during the ventricular systole, the walls of the ventricle become more convex upon the surface, the apex of the heart describes a spiral motion from behind forwards, and from right to left: in describing this spiral movement, the apex glides obliquely upon the pericardium, is approximated to the base, comes in contact with the parietes of the thorax in the intercostal space between the cartilages of the fifth and sixth ribs, and thus causes the impulse. Indeed, this part of the heart is naturally so close to the parietes of the chest, that no tilting forward of its apex is necessary to produce the slight shock felt at this period.

It was the received opinion until within a few years, that during the diastole of the ventricles, the heart receded from the parietes of the chest, and that the impulse was produced by a blow or shock given to the ribs by its apex, during the systole. Harvey, Haller, Senac, Hunter, may be quoted as authorities for this theory. The experiments which have been performed upon animals of late years, and the examination of the action of the organ in cases of ectopia of the heart, have, however, shown that this theory has no foundation, and that the heart "does not suffer any changes in consequence of its own efforts (exclusive of the movements of the lungs and diaphragm) except in its shape and size, in the thickness and tension of its parietes, and in the capacity of its cavities," which are quite sufficient to produce the slight shock felt when the hand is laid on the parietes of the chest.

M. Ritter has recently advanced this as a novel doctrine, in ignorance probably of the results of the experiments of the "Committees of the British Association." His experiments are entirely corroborative of those previously made. "The portion of the heart's surface (he observes) which is in immediate relation to the walls of the chest is at all times in close contact with them; and it is impossible that in any of its motions it can act so as to withdraw itself from the thoracic walls or so as to leave a space between them." Being thus fixed, therefore, it follows that, when the heart contracts and assumes a more globular form, it will exert its distending force on the yielding intercostal spaces against which it rests, and will thrust them forwards, so as to produce the impulse. This distending force cannot be exerted with any effect against the unyielding ribs or their cartilages; and consequently the impulse is not perceived by the finger placed over the cartilages of the fourth, fifth, or any other rib. If the impulse was caused by an actual stroke or blow of the heart against the walls of the chest, it would be perceived on these parts and on the sternum as clearly as it is in the intercostal spaces, and every person would feel the impulse of his own heart, just as a pregnant woman feels any violent movements made by the fœtus in utero."

*Sound sometimes produced by the impulse of the healthy heart.*—Although in the healthy subject, when the circulation is tranquil, and the heart's action is normal, no sound is produced by the impulse, yet it occasionally happens, that when the same heart is excited to increased action,—in other words, when palpitation ensues, whether the cause be mental emotion or corporeal exertion, but particularly the former,—the apex of the heart does come in contact with the ribs, the patient feels the blow or shock produced by the impulse of his own heart, and this is accompanied by sound, which of course will be heard at the period of the first sound of the heart, and will modify it in a certain degree, or add to it. Under such circumstances, the first sound of the heart becomes loud and ringing, and in diseased states it is sometimes so intense as to be audible without applying the stethoscope, and may be heard at a short distance from the patient. This point will be again alluded to when we come to consider the abnormal conditions of the heart.

*Seat of the impulse of the heart.*—The point at which the impulse of the heart is felt in the healthy male, is the intercostal space between the cartilages of the fifth and sixth ribs upon the left side, to the sternal side of the nipple, and about two inches below this point. In the female, owing to the habitual wearing of stays, the impulse is usually a little higher up—viz. between the cartilages of the fourth and fifth left ribs; and in the latter months of pregnancy, for an obvious reason, it is perceived still higher up, and the apex is pushed more to the left side.

The impulse of the healthy heart is naturally slight; it is more marked in the erect than in the recumbent position, because in the latter position, the heart, by its own weight, recedes slightly from the parietes of the chest. For the same reason the impulse becomes more distinct if a person leans forward, and more indistinct if he lies upon his right side. In the erect posture, the impulse is said to be slightly lower than in the recumbent posture, but the difference, if any, is very trifling. In very fat persons the impulse is scarcely perceptible to the eye or hand. In very lean persons it is very obvious to both. When the lungs are largely developed, they will overlap the heart more than usual; when the lungs are small, less of the heart will be covered by these organs: in the latter case, therefore, the impulse will be better marked than in the former.

*Alteration of the impulse in inspiration and expiration.*—In inspiration, particularly on a full inspiration, the impulse of the apex of the heart will be felt lower down than natural, as low as between the cartilages of the sixth and seventh ribs, or in the epigastrium, between the line of the xyphoid cartilage. This is partly owing to the connection of the heart with the lungs, and partly to the connection of the pericardium with the diaphragm. On a full inspiration the lungs expand from above downwards, as well as from before backwards; and, according to Dr. Sibson, from the manner in which the pulmonary veins are joined to the left auricle, the heart is drawn down by the descent of the lungs. The principal cause of this descent of the heart in inspiration, appears to lie rather in the intimate connection of the pericardium with the central tendon of the diaphragm; as the latter descends it must bring with it the heart; and from the connection of the inferior or ascending vena cava with the diaphragm, it must follow the movements of the latter, and draw down the right auricle. On a full inspiration, the impulse, in addition to being lower down, will be less marked than natural, because the lungs, when fully inflated, meet so as almost to cover the heart, and prevent its apex from coming in contact with the parietesof the thorax.

On a forced *expiration*, on the other hand, owing to the ascent of the diaphragm, the impulse of the heart is felt higher up—viz. on a line with the space between the cartilages of the fourth and fifth ribs on the left side: it is likewise more marked than natural, because the heart is less covered by lung. The point at which the impulse of the heart is felt, is altered in some diseases of this organ, or of the lungs or pleura, as well as in diseases of the abdominal viscera. These matters will, however, be considered when we come to describe the diseased states of the heart.

*Double impulse of the healthy heart.*—The impulse of the healthy heart has been almost always described as single; Magendie, however, who attributes the first sound of the heart to the shock of the apex during the ventricular systole, lays it down that the second sound is due to the shock given by the ventricles to the parietes of the thorax during their diastole. "The ventricles in dilating (he observes) in a great measure under the influence of the rapid influx of the blood, give a shock to the anterior parietes on the right side of the thorax, and thus produces the second clear sound." Dr. Sibson, in his valuable essay upon the "Changes in the Situation of the Internal Organs," observes:—"A second impulse is often felt in persons whose lungs are diminished, and whose great vessels come close to the sternum. This is synchronous with the second sound, and must, I conceive, be due to the sudden springing forwards of the walls of the right ventricle after the systole." In the year 1848, in a communication upon the subject of "Aneurism of the Aorta," made to the Surgical Society of Ireland, I called attention to the fact that the impulse of the healthy heart, when the organ is acting vigorously, is double, not single.

The impulse of the healthy heart, I observed upon that occasion, has been always described as single, just as that of aneurism of the arch of the aorta was supposed to be. If we carefully examine this organ, however, when it is beating vigoroursly, we shall find that a second but slighter impulse is perceptible, which quickly succeeds the other; and on applying the stethoscope we shall find that this second impulse accompanies the second sound of the heart: it appears as if the agency which gives rise to the second sound was capable of communicating a distinct sensation to the hand or stethoscope.

In the healthy heart the second impulse is scarcely felt, unless the organ beats vigorously; when the ventricles are somewhat hypertrophied, and their cavities somewhat dilated, the second impulse becomes better marked; when this has arrived at an extreme degree, it becomes very evident, and constitutes then the "back stroke of the heart," or the diastolic impulse. This diastolic impulse, except in cases of disease, is never so strong as to be perceptible to the eye, but is readily distinguished when the ear is applied to the stethoscope laid upon the præcordial region. It is perceived at the same part of the chest as the systolic impulse, and is more marked, the larger the surface of the heart uncovered by lung, and the stronger the action of the organ.

With respect to the cause of the diastolic impulse, Dr. Sibson observes

—" The second or diastolic impulse, which is felt between the second and third, and sometimes between the first and second costal cartilages, is neither more nor less than a sign that the upper part of the right ventricle, and the origin of the pulmonary artery, over which it is felt, are in contact with the walls of the chest." "This diastolic impulse, which is synchronous with the second sound, is a physiological, not a pathological phenomenon, and is due to the sudden return forward of the walls of the right ventricle, and of the origin of the pulmonary artery, immediately after the systole; the parts in question then impinge with a short sharp tap on the left second and third costal cartilages, and on the space between them."

When describing the motions of the heart, we saw that during ventricular diastole, the apex of the heart recedes from its base; the organ becomes elongated, the ventricles increase in all their dimensions, and the hand grasping the heart, is forcibly opened. Now, when we consider how closely the anterior surface of the ventricular portion of the heart lies to the parietes of the thorax, there is no difficulty in understanding how an impulse may be communicated during this movement, equally as during the ventricular systole ; it appears only surprising that it should have been so very generally overlooked.—*London Med. Gaz.*

